# Comparison of p53 gene mutation and protein overexpression in colorectal carcinomas.

**DOI:** 10.1038/bjc.1994.355

**Published:** 1994-10

**Authors:** B. Dix, P. Robbins, S. Carrello, A. House, B. Iacopetta

**Affiliations:** University Department of Surgery, Sir Charles Gairdner Hospital, Nedlands, Western Australia.

## Abstract

**Images:**


Br. J. Cancer (1994), 70, 585-590 © Macmillan Press Ltd., 1994

### Comparison of p53 gene mutation and protein overexpression in colorectal carcinomas

B. Dix[1], P. Robbins[2], S. Carrello[3], A. House[1] & B. Iacopetta[1]

[1]*University Department of Surgery*, [2]*University Department of Pathology, Hospital and University Pathology Services, and* [3]*Department of Histopathology, Hospital and University Pathology Services, Sir Charles Gairdner Hospital, Nedlands, Western Australia 6009.*

**Summary** Immunocytochemistry (ICC) has been used routinely to stain for p53 overexpression in a range of human tumours. The underlying assumption has been that positive staining indicates a mutation in the p53 coding sequence. Recently, however, discordancy has been observed and the accuracy of ICC as a marker of p53 gene mutation has been questioned. In this study of 109 colorectal adenocarcinomas, we compared ICC staining with p53 gene mutations detected by single-strand conformation polymorphism (SSCP) analysis. Concordancy between the two techniques was found in 69% of tumours. ICC-positive SSCP-negative cases accounted for 20% of tumours and ICC-negative SSCP-positive cases for the remaining 11%. These results caution against the assumption that p53 protein overexpression is always associated with a gene mutation. Epigenetic phenomena may account for a significant proportion of ICC-positive tumours.

p53 was originally detected as a 53 kDa nuclear phosphoprotein co-immunoprecipitating with SV40 large T antigen (Lane & Crawford, 1979; Linzer & Levine, 1979). It has since been shown to have a sequence-specific DNA-binding capability which regulates entry into the S phase of the cell cycle at a transcriptional level (Kern *et al.*, 1991). Both cytogenetic (Muleris *et al.*, 1985) and restriction fragment length polymorphism (RFLP) studies (Vogelstein *et al.*, 1989) have shown that loss of the p53 gene is a common event in human cancers. In addition to deletion of the gene, more recent studies have shown that mutation of the p53 coding sequence is also a common occurrence in a wide range of human tumours (Nigro *et al.*, 1989; Hollstein *et al.*, 1991). Alterations of the p53 gene are now known to be among the most frequent genetic changes occurring in human malignancies (Hollstein *et al.*, 1991; Caron de Fromentel & Soussi, 1992). Mutation or deletion of this gene effectively removes the regulatory influence of wild-type p53 protein on cell proliferation and may confer a growth advantage on a neoplastic population. This has led researchers to hypothesise that alterations in the p53 gene play a fundamental role in tumour development and progression.

Mutations occur most often in four of the five phylogenetically conserved regions of the p53 gene (Hollstein *et al.*, 1991; Caron de Fromentel & Soussi, 1992). When mutations occur within these regions they generally result in an altered or 'mutant' p53 protein product. Although wild-type p53 protein, because of its short half-life, is normally present at very low levels in the cell, changes in conformation brought about by mutation apparently stabilise the mutant protein (Levine *et al.*, 1991). The resultant accumulation of mutant protein may be detected by immunocytochemistry (ICC). Overexpression of the p53 protein detected by ICC has now been used in a large number of studies on various tumour types as a marker of p53 gene mutation (Bártek *et al.*, 1991; Porter *et al.*, 1992).

Although some studies have shown a good correlation between ICC staining and the presence of mutations within the p53 gene as detected by molecular analysis (Rodrigues *et al.*, 1990; Davidoff *et al.*, 1991; Marks *et al.*, 1991; Kohler *et al.*, 1992; Maestro *et al.*, 1992; Somers *et al.*, 1992; Thor *et al.*, 1992), others have revealed varying degrees of discordancy (Barton *et al.*, 1991; Allred *et al.*, 1993; Kohler *et al.*, 1993). Indeed, the use of ICC as an indicator of p53 gene mutation has been questioned (Wynford-Thomas, 1992). We undertook the present study to correlate ICC staining with p53 gene mutation in a large series of human colorectal cancers. Mutations were detected by the single-strand conformation polymorphism (SSCP) technique (Orita *et al.*, 1989) and p53 overexpression by ICC using the monoclonal antibody DO-7.

### Materials and methods

### *Tumour specimens*

A series of 109 surgically resected tumours were provided by the general surgeons at Sir Charles Gairdner Hospital. These consisted of 65 Dukes' stage B and 44 Dukes' stage C adenocarcinomas. Specimens were grossly examined and portions of tumour and adjacent normal mucosa were excised, snap frozen immediately in liquid nitrogen and stored at $-80°C$ until required for DNA extraction. The remaining tumour was fixed in neutral buffered formalin and embedded in paraffin for histopathology and ICC. The tissue specimen for SSCP analysis was taken from the centre of the tumour mass, while the tissue section for ICC was cut no more than 1-2 mm away from this site.

### *Immunocytochemistry*

Paraffin sections were cut at 5 $\mu m$, mounted on slides, dewaxed for 10 min in xylene, passed through alcohol and washed in water. They were then rinsed with 0.2 M Tris-buffered saline pH 7.6 (TBS) and endogenous peroxidase removed by soaking in 3% hydrogen peroxide for 5 min. Sections were incubated with a blocking solution of 1:5 normal swine serum and TBS for 20 min before application of the primary antibody, monoclonal mouse anti-human p53 protein (Dako p53 protein DO-7) at 1:20 dilution. The slides were incubated at room temperature overnight then washed twice with TBS before a biotinylated second antibody, anti-mouse Ig antibody (1:200) (Silenius Lab, Melbourne Australia), was applied. Streptavidin-horseradish peroxidase conjugate (Silenius Lab) was added (1:200) to all slides and incubated for 1 h before washing twice with TBS. Chromogen solution containing 6 mg of diaminobenzoate in 10 ml of TBS 30% hydrogen peroxide was then added and colour allowed to develop for 8 min before being stopped by washing in TBS. Preparations in which the primary antibody was omitted were used as negative controls. All slides were

Correspondence: B.J. Iacopetta, University Department of Surgery, Queen Elizabeth II Medical Centre, Nedlands, Western Australia 6009.
Received 5 January 1994; and in revised form 5 April 1994.

evaluated by two observers (P.R. and B.D.) and positive reactions required the presence of brown reaction product within neoplastic cells. Reactions in adjoining normal or adenomatous mucosa were also assessed. Tumours were classified as negative (no cells staining) or positive (any number of cells staining). If positive, a subjective assessment of the percentage of tumour cells staining was made.

*Preparation of genomic DNA*

Genomic DNA was prepared from frozen tissue by grinding the specimen to a powder in liquid nitrogen using a mortar and pestle. The powder was then dissolved in an extraction buffer containing 0.5% SDS, 10 mM Tris-HCl pH 8, 100 mM EDTA, 20 $\mu g\ ml^{-1}$ RNAse A and 100 $\mu g\ ml^{-1}$ proteinase K. Samples were digested overnight at 50°C on a rotating wheel apparatus and then extracted with phenol-chloroform-isoamyl alcohol (25:24:1). DNA was precipitated with one-quarter volume of 7.5 M ammonium acetate and three volumes of 100% ice-cold ethanol, washed with 70% ethanol, air dried and redissolved in 10 mM Tris-0.1 mM EDTA to a final concentration of 100 ng $\mu l^{-1}$.

*Amplification by polymerase chain reaction*

Exons 5-8 of the p53 gene were amplified separately using the following oligonucleotides (Biotech International) as primers:

| | |
|---|---|
| Exon 5 | 5'-TCTTCCTGCAGTACTCCCCT-3' |
| | 5'-AGCTGCTCACCATCGCTATC-3' |
| Exon 6 | 5'-GATTGCTCTTAGGTCTGGCC-3' |
| | 5'-GCAAACCAGACCTCAGGCGG-3' |
| Exon 7 | 5'-TTGTCTCCTAGGTTGGCTCT-3' |
| | 5'-GCTCCTGACCTGGAGTCTTC-3' |
| Exon 8 | 5'-TCCTGAGTAGTGGTAATCTA-3' |
| | 5'-GCTTGCTTACCTCGCTTAGT-3' |

The standard reaction mixture (25 $\mu l$) contained 0.4 $\mu M$ of each primer, 3 mM magnesium chloride, 67 mM Tris-HCl pH 8.8, 16.6 mM ammonium sulphate, 0.2 mM dNTPs, 0.2 $mg\ ml^{-1}$ gelatin, 0.45% Triton X-100, 0.5 $\mu Ci$ of [$\alpha$-$^{32}P$]dCTP and 0.2 units of *Taq* polymerase. Thermal cycling began with an initial denaturation of 10 min, during which time 100 ng of template DNA was added to the reaction. At least 5 min of denaturation time remained after the addition of template DNA. The initial cycle was completed with 1 min of annealing and 2 min of extension. This was followed by 35 cycles of 30 s denaturation, 1 min annealing and 2 min extension. A final extension of 10 min completed the cycling reactions. Denaturation temperature was 94°C, annealing temperatures were 62°C (exon 5), 60°C (exons 6 and 7) and 55°C (exon 8) and extension temperature was 72°C.

*SSCP analysis*

A 2 $\mu l$ volume of PCR product was denatured in 5 $\mu l$ of formamide loading buffer (95% formamide, 10 mM EDTA, 0.05% bromophenol blue and 0.05% xylene cyanol) by boiling for 10 min. Denatured samples were loaded onto 12% acrylamide-10% glycerol (99:1 acrylamide-bisacrylamide) non-denaturing gels (800 mm high × 0.4 mm thick), focused at 2,500 V for 5 min and run at 1,900 V for 20-24 h. Gels were then wrapped in Saran and exposed to X-ray film (Fuji) with intensifying screens at −80°C for 18-48 h. Samples displaying mutant bands were reamplified and run a second time to confirm the existence of a mutation. Germ-line DNA from patients displaying a mutation in their tumour DNA were also amplified and run on SSCP gels to confirm the mutation was somatically acquired.

*Sequencing of mutant bands*

Mutant bands were excised from SSCP gels using the autoradiograph as a positional template. DNA was eluted from the gel slice by immersing in 100 $\mu l$ of water overnight at room temperature, precipitated with a quarter volume of 7.5 M ammonium acetate and two volumes of 100% isopropanol, washed with 70% ethanol, air dried and resuspended in 20 $\mu l$ of 10 mM Tris-0.1 mM EDTA. The mutant DNA was sequenced by a modification of the chain termination method (Sanger *et al.*, 1977) using a Gibco BRL cycle sequencing kit. Reamplified mutant DNA was introduced as template to a set of dideoxy sequencing reactions and subjected to a repetitive series of temperature changes similar to PCR. End-labelled primer was extended by these reactions to dideoxy terminations and the sequence was obtained by separating fragments on a 6% acrylamide-7 M urea denaturing gel.

### Results

*Detection of p53 gene mutations by SSCP analysis*

A total of 109 colorectal carcinomas were screened by SSCP for mutations within exons 5-8 of the p53 gene. Mutant p53 was identified by the presence of one or two extra bands migrating above or below the normal single-stranded products (Figure 1). Occasionally mutant bands were also detected between the single- and double-stranded bands and are probably caused by normal/mutant heterodimers. In all cases, normal p53 banding patterns were also observed. Since previous work by our group has shown that p53 allelic loss occurs in 75% of these tumours (Iacopetta *et al.*, 1994), these normal bands probably arise from either an admixture of normal tissue adjoining the tumour sample and/or tumour heterogeneity. All mutations were confirmed by reamplifying samples and running on separate SSCP gels. Whenever the tumour DNA revealed a p53 mutation, the corresponding germ-line DNA was also analysed by SSCP. No germ-line mutations were found, indicating that the changes were of somatic origin.

Mutations were found in 37% (40/109) of carcinomas (Table I). The distribution of these mutations was 41% (17/41) in exon 5, 12% (5/41) in exon 6, 15% (6/41) in exon 7 and 32% (13/41) in exon 8. In one tumour (84T) two mutations were found in exon 5 and another in exon 8. Several of the most commonly occurring mutant bands detected by SSCP were sequenced and found to contain mutations at codons 175 and 248 (Table II), corresponding to two of the previously identified 'hotspot' regions (Nigro *et al.*, 1989; Hollstein *et al.*, 1991; Caron de Fromentel & Soussi, 1992). None of the mutations we sequenced was found to be a conservative basepair change. Because the primers used in this study cross the intron-exon boundary, splice donor/acceptor mutations should have been detected by SSCP analysis. However, none of the mutations we sequenced was in this region (Table II).

The sensitivity of the SSCP technique in identifying mutant DNA in the presence of large amounts of normal DNA is dependent on the resolution of the SSCP gel. Overexposure of the gel will eventually reveal all aberrantly migrating bands, providing they are sufficiently well separated from the normal bands. To determine the sensitivity of our SSCP gels, normal and mutant DNA bands from two different tumours were excised, eluted, amplified and rerun to confirm the purity of these DNA templates. They were then diluted to the same concentration and the normal and mutant alleles mixed at varying ratios from 1.5% mutant DNA to 50% mutant DNA. These mixes were amplified and run on SSCP gels (Figure 2). In cases in which the aberrantly migrating bands were well separated from the normal bands, as little as 1.5% mutant DNA in the original template mix could be detected (Figure 2a). Even when the mutant bands migrated close to normal bands, they could be detected in mixes containing 6-12% mutant DNA template (Figure 2b).

In preliminary experiments, we optimised the sensitivity of our SSCP gels by running PCR fragments from the same tumour samples under widely varying conditions of temperature, buffer concentration, glycerol/acrylamide concentration,

**Figure 1** SSCP analysis of exons 5–8 of the p53 gene. Wild-type and two tumour samples containing mutations are shown for each of the exons. Only single-stranded DNA bands are shown. Heterodimers and double-stranded bands have been omitted.

run voltage and run duration. The running conditions we selected resulted in good separation of all aberrantly migrating bands in each of the four exons.

*Detection of p53 protein overexpression by immunocytochemistry*

The anti-p53 monoclonal antibody DO-7 recognises a denaturation-resistant epitope at the N-terminus of the human p53 protein and reacts with both wild-type and mutant proteins. We used DO-7 to detect p53 overexpression by ICC staining of paraffin sections. In comparative studies using a range of antibodies, DO-7 has been shown to be one of the most sensitive for the detection of p53 overexpression (Campani *et al.*, 1993; Jiko *et al.*, 1993; Lassam *et al.*, 1993; Baas *et al.*, 1994). Overall, 46% (50/109) of colorectal carcinomas in this study exhibited positive nuclear staining (Figure 3).

**Table I** The relationship between p53 protein overexpression detected by ICC and p53 gene mutation detected by SSCP analysis of 109 colorectal carcinomas

| | *SSCP positive* | *SSCP negative* | *Total* |
|---|---|---|---|
| ICC positive | 28 (26%) | 22 (20%) | 50 (46%) |
| ICC negative | 12 (11%) | 47 (43%) | 59 (54%) |
| Total | 40 (37%) | 69 (63%) | 109 (100%) |

Two cases also displayed small amounts of reaction product in the cytoplasm of malignant cells. There was considerable heterogeneity of positive ICC reactions throughout the series and within individual tumours. The estimated percentage of positively staining neoplastic cells in given tumours ranged from 1 to 90%. No nuclear or cytoplasmic staining was detected in normal tissue which adjoined 82% (89/109) of the tumour sections.

The concordance of results between SSCP and ICC (i.e. both positive or both negative) was 69% (75/109) (Table I). A further 20% of carcinomas (22/109) stained positively by ICC but failed to display any mutation by SSCP, while the remaining 11% (12/109) displayed mutant bands by SSCP but showed no staining by ICC.

### Discussion

Mutations of the p53 gene are the most common genetic alteration, known to occur in a wide range of human cancers (Hollstein *et al.*, 1991). Under normal conditions wild-type p53 protein is rapidly degraded and is therefore present only at very low levels within the cell. The acquisition of a mutant genotype is thought to increase the half-life of the mutant protein and lead to its accumulation within the cell. This accumulated protein is detectable by immunocytochemical techniques and ICC has been proposed as an indirect method of screening tumours for mutation within the p53 gene. Recently however, a number of discordant results have been

**Table II** Characterisation of p53 mutations detected by SSCP analysis

| *Tumour no.* | *Exon* | *Codon* | *Mutation* | *Amino acid substitution* | *ICC staining*[a] |
|---|---|---|---|---|---|
| 12T | 5 | 153 | 9 bp insertion | | – |
| 19T | 7 | 242 | 4 bp insertion | | + |
| 28T | 5 | 175 | G→A | Arg → His | + |
| 38T | 5 | 175 | G→A | Arg → His | + |
| 44T | 7 | 248 | C→T | Arg → Trp | + |
| 47T | 5 | 175 | G→A | Arg → His | – |
| 54T | 5 | 175 | G→A | Arg → His | + |
| 82T | 5 | 175 | C→T | Arg → Cys | – |
| 84T[b] | 5 | 173 | Single bp deletion | | + |
| 84T[b] | 5 | 186 | Single bp deletion | | + |
| 89T | 5 | 175 | G→A | Arg → His | + |
| 90T | 5 | 152 | C→T | Pro → Leu | – |
| 95T | 5 | 175 | G→A | Arg → His | + |
| 97T | 6 | 214 | T→G | His → Gln | – |
| 112T | 7 | 254 | Single bp insertion | | + |

[a]–, negative staining by ICC; +, positive nuclear staining by ICC. [b]This tumour contained three separate mutations, two in exon 5 and one in exon 8.

**Figure 2** The sensitivity of the SSCP technique was tested by mixing mutant and normal DNA in varying proportions, amplifying and then subjecting to SSCP analysis. **a**, When mutant and normal bands were well separated, less than 1.5% mutant DNA in the initial template mix could be detected. **b**, When bands migrated more closely together approximately 6–12% mutant DNA was required for detection.

**Figure 3** Immunocytochemical staining of colorectal carcinomas with the anti-p53 antibody DO-7. Neoplastic tissue shows strong nuclear staining, whereas adjacent normal tissue shows no reaction product.

reported (Barton *et al.*, 1991; Wynford-Thomas, 1992; Allred *et al.*, 1993; Kohler *et al.*, 1993). In the present study we have attempted to address this issue by comparing p53 gene mutation and protein overexpression in a large number of colorectal tumours.

The frequency of p53 gene mutation detected by SSCP analysis in our study was 37% (40/109) in primary colorectal carcinomas, a frequency similar to recent molecular studies on large numbers of colorectal cancers (Kikuchi-Yanoshita *et al.*, 1992; Lothe *et al.*, 1992). Compared with the study by the Japanese group (Kikuchi-Yanoshita *et al.*, 1992) we found 3-fold more mutations in exon 8 and 3-fold fewer in exon 7, possibly reflecting exposure to different dietary or environmental carcinogens. As described in the results (Figure 2), we believe that the high resolution of our SSCP analysis allowed the identification of tumours containing very low levels of mutant p53 allele. For this reason we conclude that approximately 40% of colorectal carcinomas have at least some cells carrying mutations within exons 5–8 of the p53 gene (Table I).

The frequency of p53 protein overexpression detected using ICC on the same series of colorectal carcinoma specimens used for SSCP analysis was 46% (50/109). This is very similar to the range of 42–49% reported in previous ICC studies (Purdie *et al.*, 1991; Scott *et al.*, 1991; Cunningham *et al.*, 1992; Starzynska *et al.*, 1992; Sun *et al.*, 1992; Bell *et al.*, 1993). Because of the variety of methods used (different antibodies, detection systems, tissue fixation, etc.) it is often difficult to make a direct comparison between the results obtained by different groups. However, the DO-7 antibody we used appears to give the same staining profile as two other commonly used p53 antibodies, 1801 (Campani *et al.*, 1993; Lassam *et al.*, 1993) and CM-1 (Jiko *et al.*, 1993). A comparative study of six different anti-p53 antibodies has shown DO-7 to be the most sensitive and specific for ICC detection of p53 protein in colorectal cancers (Baas *et al.*, 1994).

Although the frequencies of p53 gene mutation and protein overexpression we observed were quite similar (37% and 46% respectively), the concordancy of results from the two techniques (i.e. both positive or both negative) was only 69% (75/109). Recently, Allred *et al.* (1993) reported 62% concordance between SSCP and ICC detection of p53 mutation in breast cancer. As proposed by these authors and others who have observed discrepancies between p53 gene mutation and protein overexpression (Børresen *et al.*, 1991; Wynford-Thomas, 1992; Oliner *et al.*, 1993; Slingerland *et al.*, 1993), several explanations could account for these findings. Firstly, for the group of SSCP-negative/ICC-positive tumours, (20% of cases) some mutations may have occurred outside exons 5–8. We believe that this is unlikely to account for all cases, however, especially in view of the very low frequency (<5%) of mutations known to occur outside the highly conserved regions of the p53 gene (Hollstein *et al.*, 1991; Caron de Fromentel & Soussi, 1992). Nevertheless, some of the tumours classified as SSCP negative may contain a mutation outside of exons 5–8. Masking of mutant DNA owing to the presence of large amounts of normal tissue or because of heterogeneity within individual tumours may also account for some of the SSCP-negative/ICC-positive cases. However, the SSCP technique has proven to be very sensitive in our hands, and when mutant and normal bands are well separated a mutant DNA content of as little as 1.5% is adequate for detection (Figure 2a). When mutant and normal bands were less well separated a higher mutant DNA content of about 6–12% was required for detection (Figure 2b).

In addition, the SSCP technique may not be 100% sensitive, and therefore some mutations may have escaped detection. Another explanation for SSCP-negative/ICC-positive cases is that p53 protein may accumulate in the absence of gene mutation by forming complexes with other molecules (Lane & Crawford, 1979; Sarnow *et al.*, 1982; Momand *et al.*, 1992). This sequestered protein may be functionally inactive but still detectable by p53 antibodies.

We observed 12 cases (11%) of SSCP-positive/ICC-negative tumours. The most likely explanations for these are, firstly, that the mutations do not lead to protein stabilisation and hence accumulation in the cell. Secondly, the mutated gene may code for a stop codon and lead to production of a truncated form of the protein or a halt in protein production altogether. Thirdly, the antibody used for the ICC staining may not have detected all mutant protein epitopes. We sequenced five of these 12 cases (12T, 47T, 82T, 90T and 97T) and found that four had mutations leading to amino acid substitutions and the fifth was a frameshift mutation which introduced a stop codon downstream (Table II).

Despite the 31% (34/109) discordancy, the overall correlation between SSCP and ICC detection of p53 mutations is still highly significant ($P<0.001$). Wider screening for mutations outside of the conserved gene regions and the use of other antibodies in ICC staining are likely to have uncovered other mutations which would have reduced this percentage. Nevertheless, our results suggest that ICC detection of p53 protein overexpression does not always indicate the existence of an underlying gene mutation and vice versa. This observation in colorectal cancer confirms other recent studies in large numbers of lung cancer cell lines (Bodner *et al.*, 1992) and primary breast cancers (Allred *et al.*, 1993). As suggested previously (Bodner *et al.*, 1992), overexpression of p53 protein may depend upon the type of gene mutation.

An obvious question which arises from this work is the comparative biological significance, if any, of p53 gene mutations and protein overexpression in neoplasia. It has been suggested that ICC detects functionally significant mutations (Wynford-Thomas, 1992). Certainly, in breast cancer, overexpression of p53 appears to be a significant marker of poor prognosis (Isola *et al.*, 1992; Thor *et al.*, 1992; Allred *et al.*, 1993; Silvestrini *et al.*, 1993). In colorectal cancer, however, the association between ICC positivity and patient survival is still controversial (Scott *et al.*, 1991; O'Connell *et al.*, 1992; Remvikos *et al.*, 1992; Starzynska *et al.*, 1992; Sun *et al.*, 1992; Yamaguchi *et al.*, 1992; Bell *et al.*, 1993). Much less work has been done correlating p53 gene mutation and outcome. Two studies in breast cancer indicate poorer prognosis in the presence of p53 gene mutation (Allred *et al.*, 1993; Thorlacius *et al.*, 1993), but to our knowledge none has been published so far on colorectal carcinoma. Although the post-operative follow-up time of patients whose tumours were examined in the current study is still quite short (average 30 months), our preliminary results show no significant correlation between p53 gene mutation and survival (Dix *et al.*, 1994).

This work was supported by grants from the Cancer Foundation of Western Australia, The Sir Charles Gairdner Hospital Research Foundation and the Faculty of Medicine, University of Western Australia. The authors gratefully acknowledge the cooperation of the general surgeons (R. Bell, G. Clarke, G. Cullingford, K. Faulkner, D. Ingram, D. Kermode, A. Kierath, M. Levitt and P. Smith) at the Sir Charles Gairdner Hospital for the provision of tumour specimens.

### References

ALLRED, D.C., CLARK, G.M., ELLEDGE, R., FUQUA, S.A.W., BROWN, R.W., CHAMNESS, G.C., OSBORNE, C.K. & McGUIRE, W.L. (1993). Association of p53 protein expression with tumor cell proliferation rate and clinical outcome in node-negative breast cancer. *J. Natl Cancer Inst.*, **85**, 200-206.

BAAS, I.O., MULDER, J.-W.R., OFFERHAUS, G.J.A., VOGELSTEIN, B. & HAMILTON, S.R. (1994). An evaluation of six antibodies for immunohistochemistry of mutant p53 gene product in archival colorectal neoplasms. *J. Pathol.*, **172**, 5-12.

BÁRTEK, J., BÁRTKOVÁ, J., VOJTESEK, B., STASKOVÁ, Z., LUKÁS, J., REJTHAR, A., KOVARIK, J., MIDGLEY, C.A., GANNON, J.V. & LANE, D.P. (1991). Aberrant expression of the p53 oncoprotein is a common feature of a wide spectrum of human malignancies. *Oncogene*, **6**, 1699-1703.

BARTON, C.M., STADDON, S.L., HUGHES, C.M., HALL, P.A., O'SULLIVAN, C., KLÖPPEL, G., THEIS, B., RUSSELL, R.C.G., NEOPTOLEMOS, J., WILLIAMSON, R.C.N., LANE, D.P. & LEMOINE, N.R. (1991). Abnormalities of the p53 tumour suppressor gene in human pancreatic cancer. *Br. J. Cancer*, **64**, 1076-1082.

BELL, S.M., SCOTT, N., CROSS, D., SAGAR, P., LEWIS, F.A., BLAIR, G.R., TAYLOR, G.R., DIXON, M.F. & QUIRKE, P. (1993). Prognostic value of p53 overexpression and c-Ki-ras gene mutations in colorectal cancer. *Gastroenterology*, **104**, 57-64.

BODNER, S.M., MINNA, J.D., JENSEN, S.M., D'AMICO, D., CARBONE, D., MITSUDOMI, T., FEDORKO, J., BUCHAGEN, D.L., NAU, M.M., GAZDAR, A.F. & LINNOILA, R.I. (1992). Expression of mutant p53 proteins in lung cancer correlates with the class of p53 gene mutation. *Oncogene*, **7**, 743-749.

BØRRESEN, A.-L., HOVIG, E., SMITH-SØRENSEN, B., MALKIN, D., LYSTAD, S., ANDERSEN, T.I., NESLAND, J.M., ISSELBACHER, K.J. & FRIEND, S.H. (1991). Constant denaturant gel electrophoresis as a rapid screening technique for p53 mutations. *Proc. Natl Acad. Sci. USA*, **88**, 8405-8409.

CAMPANI, D., CECCHETTI, D. & BEVILACQUA, G. (1993). Immunocytochemical p53 detection by microwave oven heating of routinely formalin-fixed paraffin sections. *J. Pathol.*, **171**, 151-152.

CARON DE FROMENTEL, C. & SOUSSI, T. (1992). TP53 tumor suppressor gene: a model for investigating human mutagenesis. *Genes Chrom. Cancer*, **4**, 1-15.

CUNNINGHAM, J., LUST, J.A., SCHAID, D.J., BREN, G.D., CARPENTER, H.A., RIZZA, E., KOVACH, J.S. & THIBODEAU, S.N. (1992). Expression of *p53* and 17p allelic loss in colorectal carcinoma. *Cancer Res.*, **52**, 1974-1980.

DAVIDOFF, A.M., HUMPHREY, P.A., IGLEHART, J.D. & MARKS, J.R. (1991). Genetic basis for p53 overexpression in human breast cancer. *Proc. Natl Acad. Sci. USA*, **88**, 5006-5010.

DIX, B.R., ROBBINS, P., SOONG, R., JENNER, D., HOVE, A.K., IACOPETTA, B.J. AND THE GENERAL SURGEONS AT SIR CHARLES GAIRDNER HOSPITAL (1994). The common molecular genetic alteration in Dukes' B and C colorectal carcinomas are not short-term prognostic indications of survival. *Int. J. Cancer* (in press).

HOLLSTEIN, M., SIDRANSKY, D., VOGELSTEIN, B. & HARRIS, C.C. (1991). p53 mutations in human cancers. *Science*, **253**, 49-53.

IACOPETTA, B., DIGRANDI, S., DIX, B., HAIG, C., SOONG, R. & HOUSE, A. (1994). Loss of tumour suppressor gene loci in human colorectal carcinoma. *Eur. J. Cancer* (in press).

ISOLA, J., VISAKORPI, T., HOLLI, K. & KALLIONIEMI, O.P. (1992). Association of overexpression of tumor suppressor protein p53 with rapid cell proliferation and poor prognosis in node-negative breast cancer patients. *J. Natl Cancer Inst.*, **84**, 1109-1114.

JIKO, K., SASANO, H., ITO, K., OZAWA, N., SATO, S. & YAJIMA, A. (1993). Immunohistochemical and *in situ* hybridization analysis of p53 in human endometrial carcinoma of the uterus. *Anticancer Res.*, **13**, 305-310.

KERN, S.E., KINZLER, K.W., BRUSKIN, A., JAROSZ, D., FRIEDMAN, P., PRIVES, C. & VOGELSTEIN, B. (1991). Identification of p53 as a sequence-specific DNA-binding protein. *Science*, **252**, 1708-1711.

KIKUCHI-YANOSHITA, R., KONISHI, M., ITO, S., SEKI, M., TANAKA, K., MAEDA, Y., IINO, H., FUKAYAMA, M., KOIKE, M., MORI, T., SAKURABA, H., FUKUNARI, H., IWAMA, T. & MIYAKI, M. (1992). Genetic changes of both p53 alleles associated with the conversion from colorectal adenoma to early carcinoma in familial adenomatous polyposis and non-familial adenomatous polyposis patients. *Cancer Res.*, **52**, 3965-3971.

KOHLER, M.F., BERCHUCK, A., DAVIDOFF, A.M., HUMPHREY, P.A., DODGE, R.K., IGLEHART, J.D., SOPER, J.T., CLARKE-PEARSON, D.L., BAST, R.C.J. & MARKS, J.R. (1992). Overexpression and mutation of p53 in endometrial carcinoma. *Cancer Res.*, **52**, 1622-1627.

KOHLER, M.F., KERNS, B.-J.M., HUMPHREY, P.A., MARKS, J.R., BAST, R.C.J. & BERCHUCK, A. (1993). Mutation and overexpression of p53 in early-stage epithelial ovarian cancer. *Obstet. Gynecol.*, **81**, 643-650.

LANE, D.P. & CRAWFORD, L.V. (1979). T antigen is bound to a host protein in SV40-transformed cells. *Nature*, **278**, 261-263.

LASSAM, N.J., FROM, L. & KAHN, H.J. (1993). Overexpression of p53 is a late event in the development of malignant melanoma. *Cancer Res.*, **53**, 2235-2238.

LEVINE, A.J., MOMAND, J. & FINLAY, C.A. (1991). The p53 tumour suppressor gene. *Nature*, **351**, 453-456.

LINZER, D.I.H. & LEVINE, A.J. (1979). Characterization of a 54K dalton cellular SV40 tumor antigen present in SV40-transformed cells and uninfected embryonal carcinoma cells. *Cell*, **17**, 43-52.

LOTHE, R.A., FOSSLI, T., DANIELSEN, H.E., STENWIG, A.E., NESLAND, J.M., GALLIE, B. & BØRRESEN, A.-L. (1992). Molecular genetic studies of tumor suppressor gene regions on chromosomes 13 and 17 in colorectal tumors. *J. Natl Cancer Inst.*, **84**, 1100-1108.

MAESTRO, R., DOLCETTI, R., GASPAROTTO, D., DOGLIONI, C., PELUCCHI, S., BARZAN, L., GRANDI, E. & BOIOCCHI, M. (1992). High frequency of p53 gene alterations associated with protein overexpression in human squamous cell carcinoma of the larynx. *Oncogene*, **7**, 1159-1166.

MARKS, J.R., DAVIDOFF, A.M., KERNS, B.J., HUMPHREY, P.A., PENCE, J.C., DODGE, R.K., CLARKE-PEARSON, D.L., IGLEHART, J.D., BAST, R.C.J. & BERCHUCK, A. (1991). Overexpression and mutation of p53 in epithelial ovarian cancer. *Cancer Res.*, **51**, 2979-2984.

MOMAND, J., ZAMBETTI, G.P., OLSON, D.C., GEORGE, D. & LEVINE, A.J. (1992). The mdm-2 oncogene product forms a complex with the p53 protein and inhibits p53-mediated transactivation. *Cell*, **69**, 1237-1245.

MULERIS, M., SALMON, R.J., ZAFRANI, B., GIRODET, J. & DUTRILLAUX, B. (1985). Consistent deficiencies of chromosome 18 and of the short arm of chromosome 17 in eleven cases of human large bowel cancer: a possible recessive determinism. *Ann. Genet.*, **28**, 206-213.

NIGRO, J.M., BAKER, S.J., PREISINGER, A.C., JESSUP, J.M., HOSTETTER, R., CLEARY, K., BIGNER, S.H., DAVIDSON, N., BAYLIN, S., DEVILEE, P., GLOVER, T., COLLINS, F.S., WESTON, A., MODALI, R., HARRIS, C.C. & VOGELSTEIN, B. (1989). Mutations in the p53 gene occur in diverse human tumour types. *Nature*, **342**, 705-708.

O'CONNELL, M.J., SCHAID, D.J., GANJU, V., CUNNINGHAM, J., KOVACH, J.S. & THIBODEAU, S.N. (1992). Current status of adjuvant chemotherapy for colorectal cancer. *Cancer*, **70**, 1732-1739.

OLINER, J.D., PIETENPOL, J.A., THIAGALINGAM, S., GYURIS, J., KINZLER, K.W. & VOGELSTEIN, B. (1993). Oncoprotein MDM2 conceals the activation domain of tumour suppressor p53. *Nature*, **362**, 857-860.

ORITA, M., SUZUKI, Y., SEKIYA, T. & HAYASHI, K. (1989). Rapid and sensitive detection of point mutations and DNA polymorphisms using the polymerase chain reaction. *Genomics*, **5**, 874-879.

PORTER, P.L., GOWN, A.M., KRAMP, S.G. & COLTRERA, M.D. (1992). Widespread p53 overexpression in human malignant tumors. *Am. J. Pathol.*, **140**, 145-153.

PURDIE, C.A., O'GRADY, J., PIRIS, J., WYLLIE, A.H. & BIRD, C.C. (1991). p53 expression in colorectal tumors. *Am. J. Pathol.*, **138**, 807-813.

REMVIKOS, Y., TOMINAGA, O., HAMMEL, P., LAURENT-PUIG, P., SALMON, R.J., DUTRILLAUX, B. & THOMAS, G. (1992). Increased p53 protein content of colorectal tumours correlates with poor survival. *Br. J. Cancer*, **66**, 758-764.

RODRIGUES, N.R., ROWAN, A., SMITH, M.E.F., KERR, I.B., BODMER, W.F., GANNON, J.V. & LANE, D.P. (1990). p53 mutations in colorectal cancer. *Proc. Natl Acad. Sci. USA*, **87**, 7555-7559.

SANGER, F., NICKLEN, S. & COULSON, A.R. (1977). DNA sequencing with chain-terminating inhibitors. *Proc. Natl Acad. Sci. USA*, **74**, 5463-5467.

SARNOW, P., HO, Y.S., WILLIAMS, J. & LEVINE, A.J. (1982). Adenovirus E1B-58kD tumour antigen and SV-40 large tumour antigen are physically associated with the same 54 kD cellular protein in transformed cells. *Cell*, **28**, 387-394.

SCOTT, N., SAGAR, P., STEWART, J., BLAIR, G.E., DIXON, M.F. & QUIRKE, P. (1991). p53 in colorectal cancer: clinicopathological correlation and prognostic significance. *Br. J. Cancer*, **63**, 317-319.

SILVESTRINI, R., BENINI, E., DAIDONE, M.G., VENERONI, S., BORACCHI, P., CAPPELLETTI, V., DI FRONZO, G. & VERONESI, U. (1993). p53 as an independent prognostic marker in lymph node-negative breast cancer patients. *J. Natl Cancer Inst.*, **85**, 965-970.

SLINGERLAND, J.M., JENKINS, J.R. & BENCHIMOL, S. (1993). The transforming and suppressor functions of p53 alleles: effects of mutations that disrupt phosphorylation, oligomerization and nuclear translocation. *EMBO J.*, **12**, 1029-1037.

SOMERS, K.D., MERRICK, M.A., LOPEZ, M.E., INCOGNITO, L.S., SCHECHTER, G.L. & CASEY, G. (1992). Frequent p53 mutations in head and neck cancer. *Cancer Res.*, **52**, 5997-6000.

STARZYNSKA, T., BROMLEY, M., GHOSH, A. & STERN, P.L. (1992). Prognostic significance of p53 overexpression in gastric and colorectal carcinoma. *Br. J. Cancer*, **66**, 558-562.

SUN, X.-F., CARSTENSEN, J.M., ZHANG, H., STAL, O., WINGREN, S., HATSCHEK, T. & NORDENSKJÖLD, B. (1992). Prognostic significance of cytoplasmic p53 oncoprotein in colorectal adenocarcinoma. *Lancet*, **340**, 1369-1373.

THOR, A.D., MOORE II, D.H., EDGERTON, S.M., KAWASAKI, E.S., REIHSAUS, E., LYNCH, H.T., MARCUS, J.N., SCHWARTZ, L., CHEN, L.-C., MAYALL, B.H. & SMITH, H.S. (1992). Accumulation of p53 tumor suppressor gene protein: an independent marker of prognosis in breast cancers. *J. Natl Cancer Inst.*, **84**, 845-855.

THORLACIUS, S., BÖRRESEN, A.-L. & EYFJÖRD, J.E. (1993). Somatic *p53* mutations in human breast carcinomas in an Icelandic population: a prognostic factor. *Cancer Res.*, **53**, 1637-1641.

VOGELSTEIN, B., FEARON, E.R., KERN, S.E., HAMILTON, S.R., PREISINGER, A.C., NAKAMURA, Y. & WHITE, R. (1989). Allelotype of colorectal carcinomas. *Science*, **244**, 207-211.

WYNFORD-THOMAS, D. (1992). p53 in tumour pathology: can we trust immunocytochemistry? *J. Pathol*, **166**, 329-330.

YAMAGUCHI, A., KUROSAKA, Y., FUSHIDA, S., KANNO, M., YONEMURA, Y., MIWA, K. & MIYAZAKI, I. (1992). Expression of p53 protein in colorectal cancer and its relationship to short-term prognosis. *Cancer*, **70**, 2778-2784.


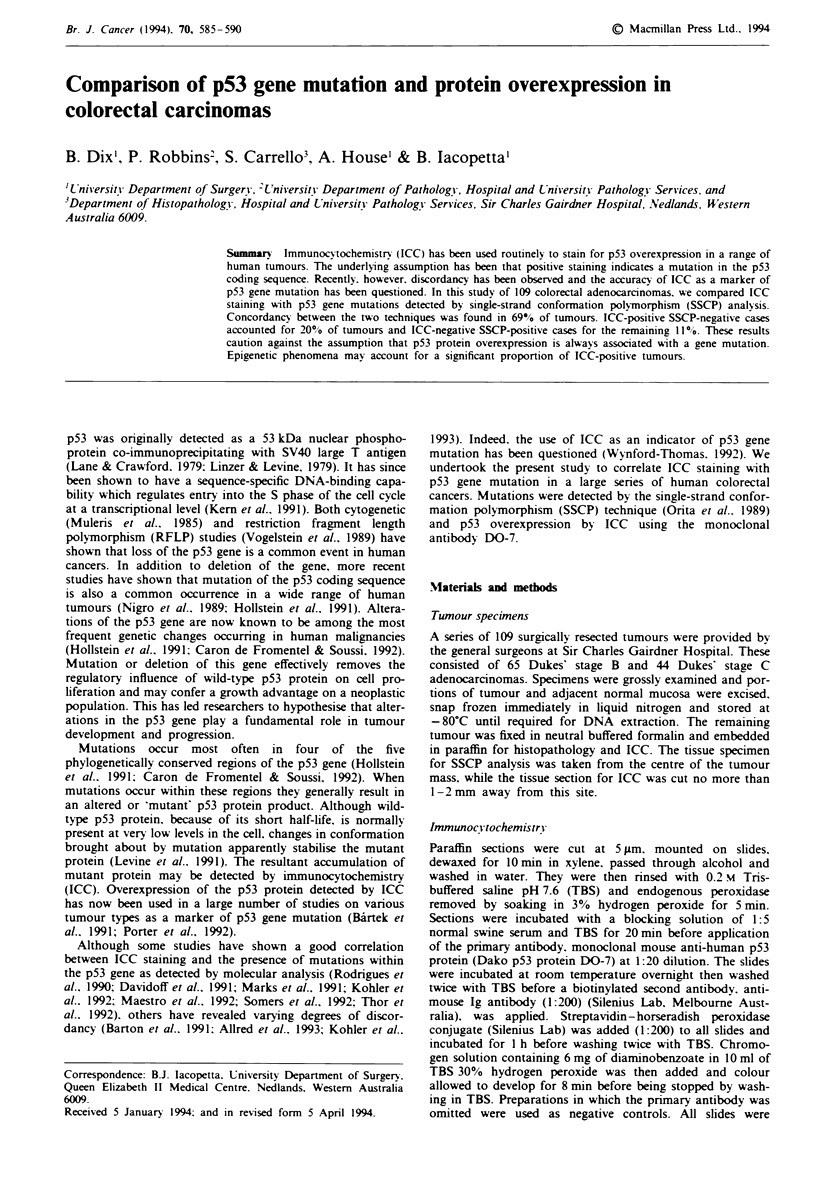

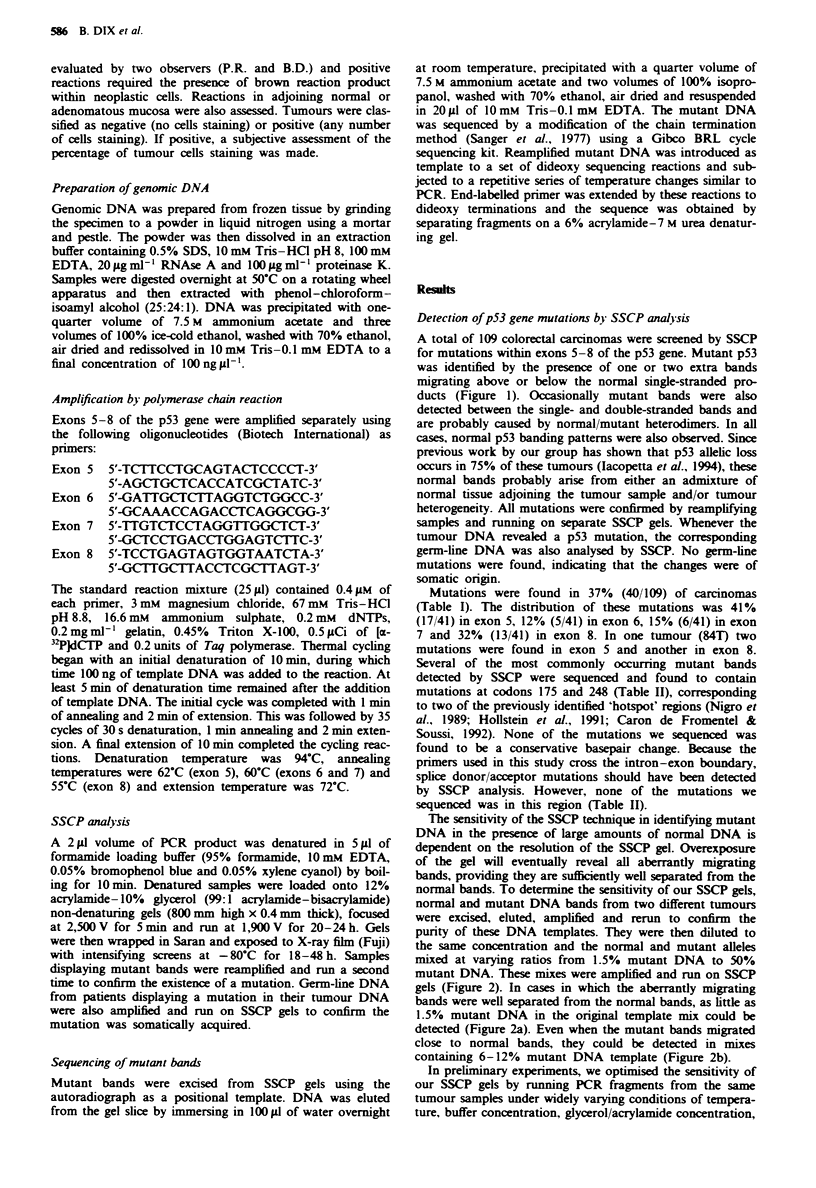

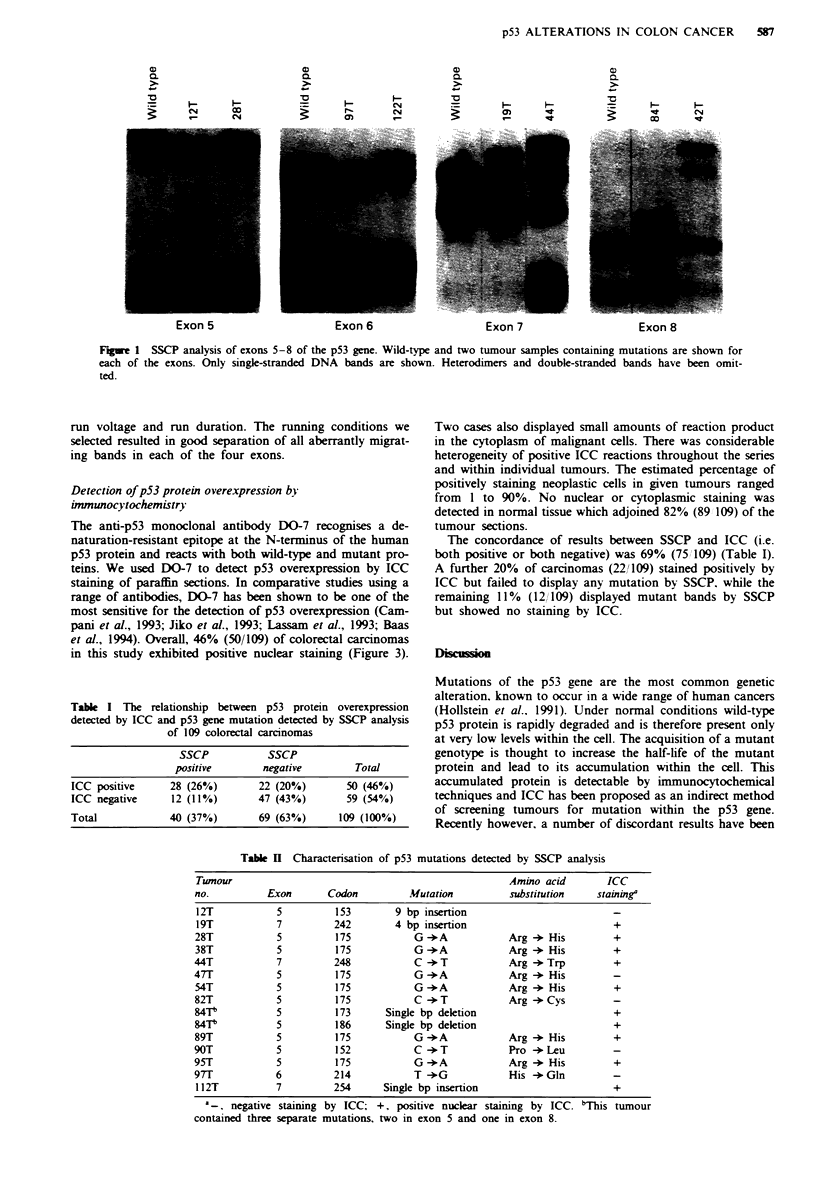

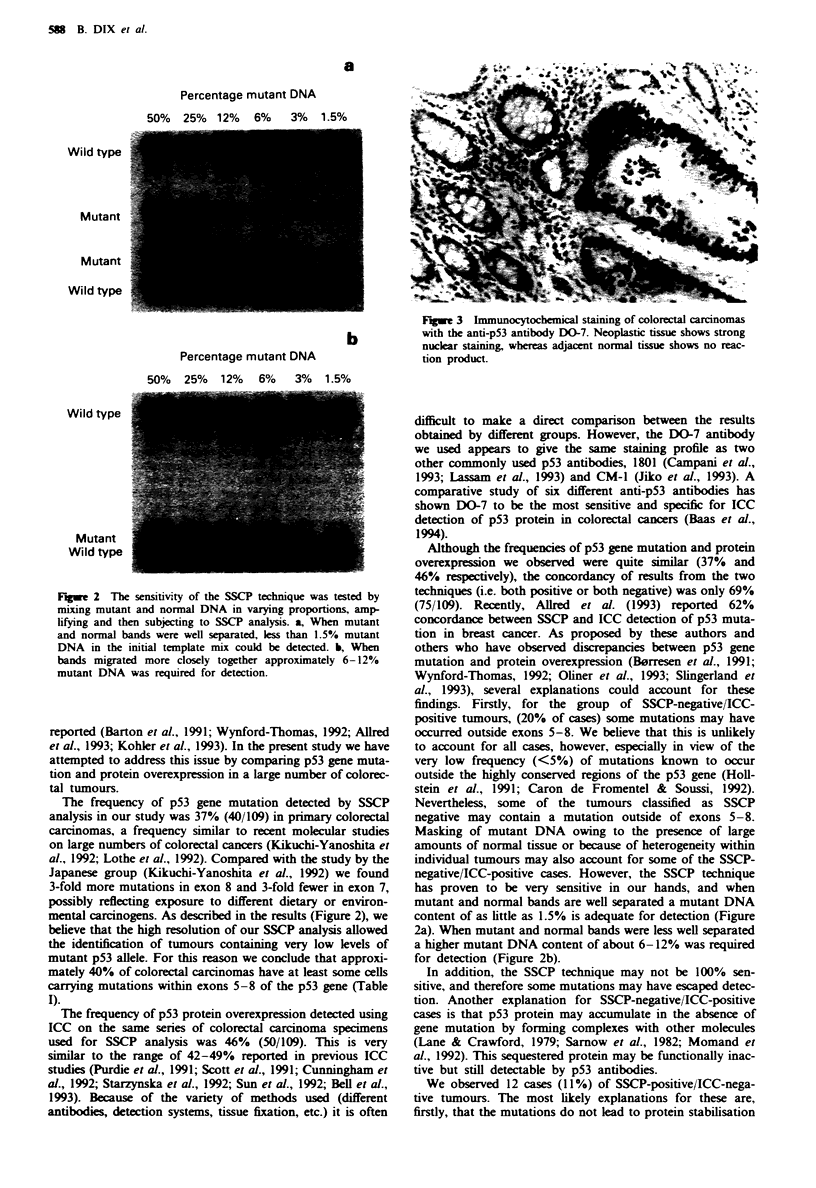

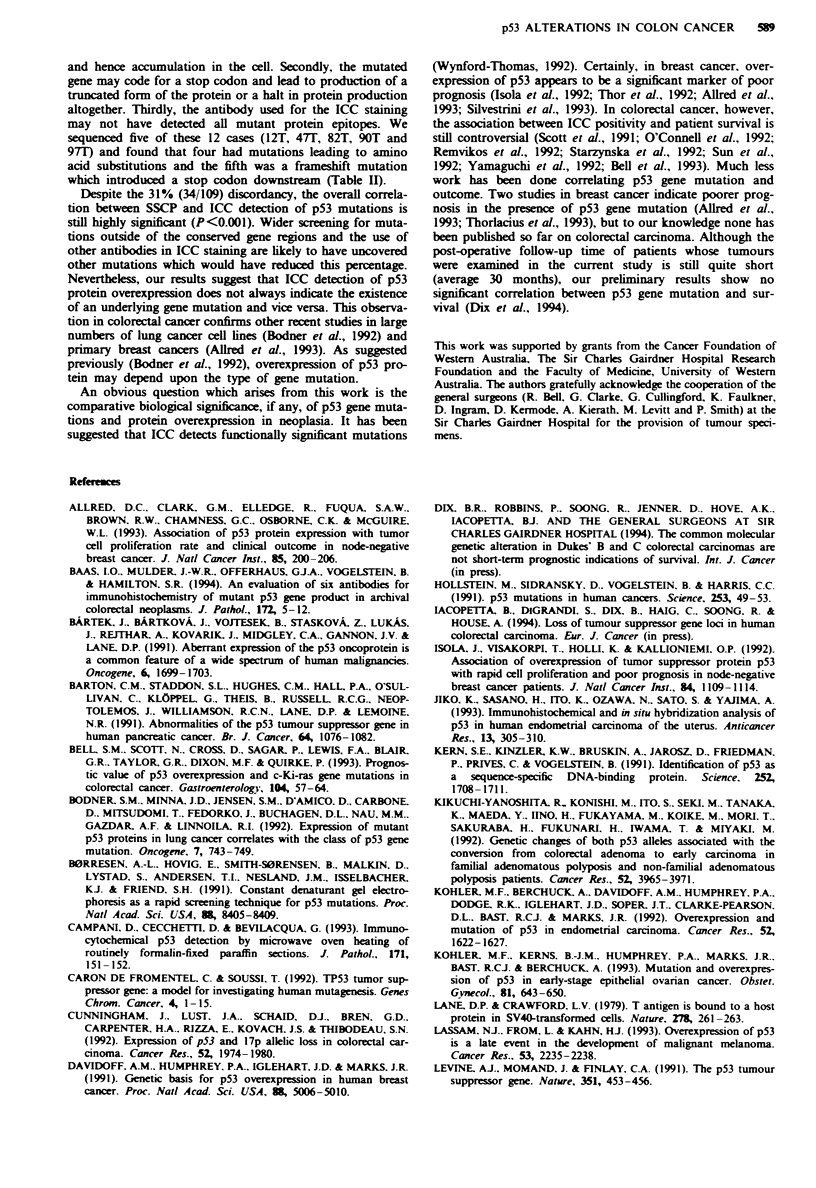

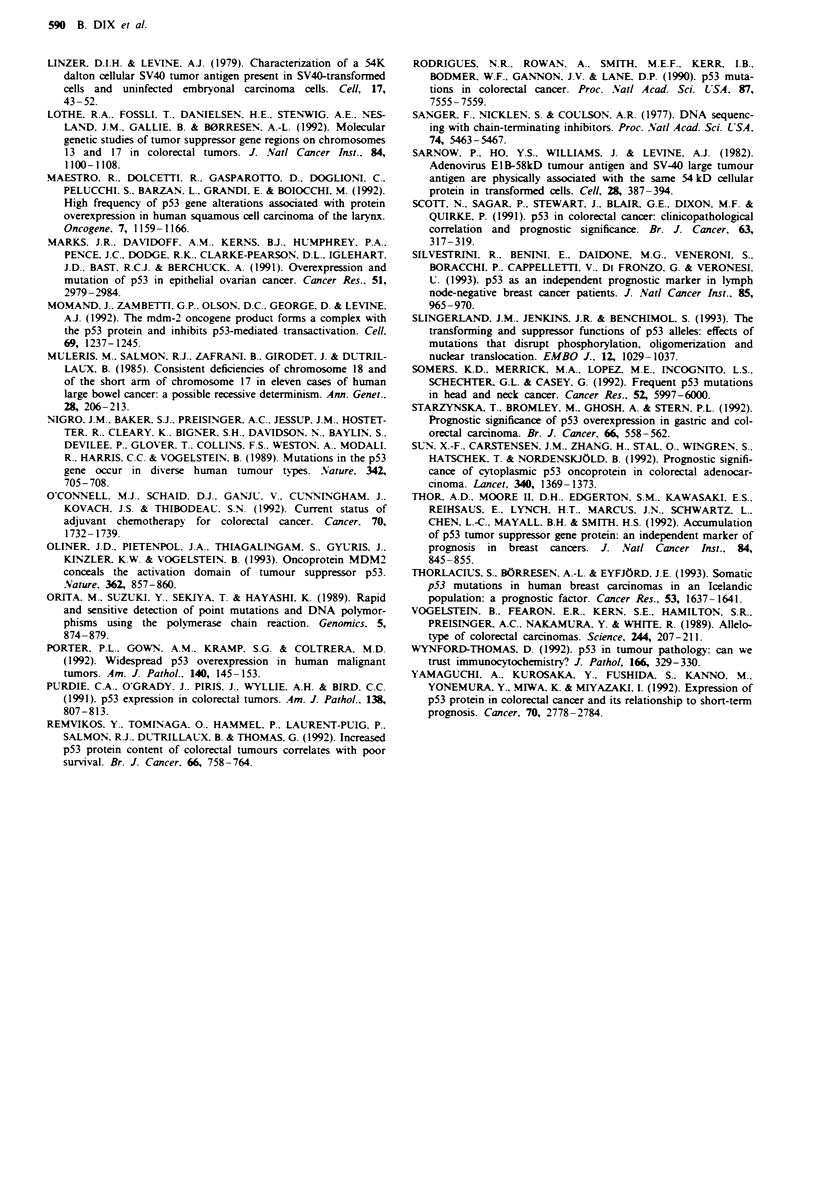


## References

[OCR_00612] Allred D. C., Clark G. M., Elledge R., Fuqua S. A., Brown R. W., Chamness G. C., Osborne C. K., McGuire W. L. (1993). Association of p53 protein expression with tumor cell proliferation rate and clinical outcome in node-negative breast cancer.. J Natl Cancer Inst.

[OCR_00617] Baas I. O., Mulder J. W., Offerhaus G. J., Vogelstein B., Hamilton S. R. (1994). An evaluation of six antibodies for immunohistochemistry of mutant p53 gene product in archival colorectal neoplasms.. J Pathol.

[OCR_00632] Barton C. M., Staddon S. L., Hughes C. M., Hall P. A., O'Sullivan C., Klöppel G., Theis B., Russell R. C., Neoptolemos J., Williamson R. C. (1991). Abnormalities of the p53 tumour suppressor gene in human pancreatic cancer.. Br J Cancer.

[OCR_00637] Bell S. M., Scott N., Cross D., Sagar P., Lewis F. A., Blair G. E., Taylor G. R., Dixon M. F., Quirke P. (1993). Prognostic value of p53 overexpression and c-Ki-ras gene mutations in colorectal cancer.. Gastroenterology.

[OCR_00646] Bodner S. M., Minna J. D., Jensen S. M., D'Amico D., Carbone D., Mitsudomi T., Fedorko J., Buchhagen D. L., Nau M. M., Gazdar A. F. (1992). Expression of mutant p53 proteins in lung cancer correlates with the class of p53 gene mutation.. Oncogene.

[OCR_00626] Bártek J., Bártková J., Vojtesek B., Stasková Z., Lukás J., Rejthar A., Kovarík J., Midgley C. A., Gannon J. V., Lane D. P. (1991). Aberrant expression of the p53 oncoprotein is a common feature of a wide spectrum of human malignancies.. Oncogene.

[OCR_00650] Børresen A. L., Hovig E., Smith-Sørensen B., Malkin D., Lystad S., Andersen T. I., Nesland J. M., Isselbacher K. J., Friend S. H. (1991). Constant denaturant gel electrophoresis as a rapid screening technique for p53 mutations.. Proc Natl Acad Sci U S A.

[OCR_00657] Campani D., Cecchetti D., Bevilacqua G. (1993). Immunocytochemical p53 detection by microwave oven heating of routinely formalin-fixed paraffin sections.. J Pathol.

[OCR_00663] Caron de Fromentel C., Soussi T. (1992). TP53 tumor suppressor gene: a model for investigating human mutagenesis.. Genes Chromosomes Cancer.

[OCR_00670] Cunningham J., Lust J. A., Schaid D. J., Bren G. D., Carpenter H. A., Rizza E., Kovach J. S., Thibodeau S. N. (1992). Expression of p53 and 17p allelic loss in colorectal carcinoma.. Cancer Res.

[OCR_00676] Davidoff A. M., Humphrey P. A., Iglehart J. D., Marks J. R. (1991). Genetic basis for p53 overexpression in human breast cancer.. Proc Natl Acad Sci U S A.

[OCR_00687] Hollstein M., Sidransky D., Vogelstein B., Harris C. C. (1991). p53 mutations in human cancers.. Science.

[OCR_00697] Isola J., Visakorpi T., Holli K., Kallioniemi O. P. (1992). Association of overexpression of tumor suppressor protein p53 with rapid cell proliferation and poor prognosis in node-negative breast cancer patients.. J Natl Cancer Inst.

[OCR_00703] Jiko K., Sasano H., Ito K., Ozawa N., Sato S., Yajima A. (1993). Immunohistochemical and in situ hybridization analysis of p53 in human endometrial carcinoma of the uterus.. Anticancer Res.

[OCR_00710] Kern S. E., Kinzler K. W., Bruskin A., Jarosz D., Friedman P., Prives C., Vogelstein B. (1991). Identification of p53 as a sequence-specific DNA-binding protein.. Science.

[OCR_00713] Kikuchi-Yanoshita R., Konishi M., Ito S., Seki M., Tanaka K., Maeda Y., Iino H., Fukayama M., Koike M., Mori T. (1992). Genetic changes of both p53 alleles associated with the conversion from colorectal adenoma to early carcinoma in familial adenomatous polyposis and non-familial adenomatous polyposis patients.. Cancer Res.

[OCR_00722] Kohler M. F., Berchuck A., Davidoff A. M., Humphrey P. A., Dodge R. K., Iglehart J. D., Soper J. T., Clarke-Pearson D. L., Bast R. C., Marks J. R. (1992). Overexpression and mutation of p53 in endometrial carcinoma.. Cancer Res.

[OCR_00729] Kohler M. F., Kerns B. J., Humphrey P. A., Marks J. R., Bast R. C., Berchuck A. (1993). Mutation and overexpression of p53 in early-stage epithelial ovarian cancer.. Obstet Gynecol.

[OCR_00735] Lane D. P., Crawford L. V. (1979). T antigen is bound to a host protein in SV40-transformed cells.. Nature.

[OCR_00739] Lassam N. J., From L., Kahn H. J. (1993). Overexpression of p53 is a late event in the development of malignant melanoma.. Cancer Res.

[OCR_00744] Levine A. J., Momand J., Finlay C. A. (1991). The p53 tumour suppressor gene.. Nature.

[OCR_00750] Linzer D. I., Levine A. J. (1979). Characterization of a 54K dalton cellular SV40 tumor antigen present in SV40-transformed cells and uninfected embryonal carcinoma cells.. Cell.

[OCR_00756] Lothe R. A., Fossli T., Danielsen H. E., Stenwig A. E., Nesland J. M., Gallie B., Børresen A. L. (1992). Molecular genetic studies of tumor suppressor gene regions on chromosomes 13 and 17 in colorectal tumors.. J Natl Cancer Inst.

[OCR_00763] Maestro R., Dolcetti R., Gasparotto D., Doglioni C., Pelucchi S., Barzan L., Grandi E., Boiocchi M. (1992). High frequency of p53 gene alterations associated with protein overexpression in human squamous cell carcinoma of the larynx.. Oncogene.

[OCR_00774] Marks J. R., Davidoff A. M., Kerns B. J., Humphrey P. A., Pence J. C., Dodge R. K., Clarke-Pearson D. L., Iglehart J. D., Bast R. C., Berchuck A. (1991). Overexpression and mutation of p53 in epithelial ovarian cancer.. Cancer Res.

[OCR_00779] Momand J., Zambetti G. P., Olson D. C., George D., Levine A. J. (1992). The mdm-2 oncogene product forms a complex with the p53 protein and inhibits p53-mediated transactivation.. Cell.

[OCR_00785] Muleris M., Salmon R. J., Zafrani B., Girodet J., Dutrillaux B. (1985). Consistent deficiencies of chromosome 18 and of the short arm of chromosome 17 in eleven cases of human large bowel cancer: a possible recessive determinism.. Ann Genet.

[OCR_00790] Nigro J. M., Baker S. J., Preisinger A. C., Jessup J. M., Hostetter R., Cleary K., Bigner S. H., Davidson N., Baylin S., Devilee P. (1989). Mutations in the p53 gene occur in diverse human tumour types.. Nature.

[OCR_00798] O'Connell M. J., Schaid D. J., Ganju V., Cunningham J., Kovach J. S., Thibodeau S. N. (1992). Current status of adjuvant chemotherapy for colorectal cancer. Can molecular markers play a role in predicting prognosis?. Cancer.

[OCR_00806] Oliner J. D., Pietenpol J. A., Thiagalingam S., Gyuris J., Kinzler K. W., Vogelstein B. (1993). Oncoprotein MDM2 conceals the activation domain of tumour suppressor p53.. Nature.

[OCR_00810] Orita M., Suzuki Y., Sekiya T., Hayashi K. (1989). Rapid and sensitive detection of point mutations and DNA polymorphisms using the polymerase chain reaction.. Genomics.

[OCR_00816] Porter P. L., Gown A. M., Kramp S. G., Coltrera M. D. (1992). Widespread p53 overexpression in human malignant tumors. An immunohistochemical study using methacarn-fixed, embedded tissue.. Am J Pathol.

[OCR_00823] Purdie C. A., O'Grady J., Piris J., Wyllie A. H., Bird C. C. (1991). p53 expression in colorectal tumors.. Am J Pathol.

[OCR_00826] Remvikos Y., Tominaga O., Hammel P., Laurent-Puig P., Salmon R. J., Dutrillaux B., Thomas G. (1992). Increased p53 protein content of colorectal tumours correlates with poor survival.. Br J Cancer.

[OCR_00832] Rodrigues N. R., Rowan A., Smith M. E., Kerr I. B., Bodmer W. F., Gannon J. V., Lane D. P. (1990). p53 mutations in colorectal cancer.. Proc Natl Acad Sci U S A.

[OCR_00838] Sanger F., Nicklen S., Coulson A. R. (1977). DNA sequencing with chain-terminating inhibitors.. Proc Natl Acad Sci U S A.

[OCR_00845] Sarnow P., Ho Y. S., Williams J., Levine A. J. (1982). Adenovirus E1b-58kd tumor antigen and SV40 large tumor antigen are physically associated with the same 54 kd cellular protein in transformed cells.. Cell.

[OCR_00852] Scott N., Sagar P., Stewart J., Blair G. E., Dixon M. F., Quirke P. (1991). p53 in colorectal cancer: clinicopathological correlation and prognostic significance.. Br J Cancer.

[OCR_00855] Silvestrini R., Benini E., Daidone M. G., Veneroni S., Boracchi P., Cappelletti V., Di Fronzo G., Veronesi U. (1993). p53 as an independent prognostic marker in lymph node-negative breast cancer patients.. J Natl Cancer Inst.

[OCR_00862] Slingerland J. M., Jenkins J. R., Benchimol S. (1993). The transforming and suppressor functions of p53 alleles: effects of mutations that disrupt phosphorylation, oligomerization and nuclear translocation.. EMBO J.

[OCR_00868] Somers K. D., Merrick M. A., Lopez M. E., Incognito L. S., Schechter G. L., Casey G. (1992). Frequent p53 mutations in head and neck cancer.. Cancer Res.

[OCR_00873] Starzynska T., Bromley M., Ghosh A., Stern P. L. (1992). Prognostic significance of p53 overexpression in gastric and colorectal carcinoma.. Br J Cancer.

[OCR_00880] Sun X. F., Carstensen J. M., Zhang H., Stål O., Wingren S., Hatschek T., Nordenskjöld B. (1992). Prognostic significance of cytoplasmic p53 oncoprotein in colorectal adenocarcinoma.. Lancet.

[OCR_00884] Thor A. D., Moore DH I. I., Edgerton S. M., Kawasaki E. S., Reihsaus E., Lynch H. T., Marcus J. N., Schwartz L., Chen L. C., Mayall B. H. (1992). Accumulation of p53 tumor suppressor gene protein: an independent marker of prognosis in breast cancers.. J Natl Cancer Inst.

[OCR_00892] Thorlacius S., Börresen A. L., Eyfjörd J. E. (1993). Somatic p53 mutations in human breast carcinomas in an Icelandic population: a prognostic factor.. Cancer Res.

[OCR_00897] Vogelstein B., Fearon E. R., Kern S. E., Hamilton S. R., Preisinger A. C., Nakamura Y., White R. (1989). Allelotype of colorectal carcinomas.. Science.

[OCR_00902] Wynford-Thomas D. (1992). P53 in tumour pathology: can we trust immunocytochemistry?. J Pathol.

[OCR_00906] Yamaguchi A., Kurosaka Y., Fushida S., Kanno M., Yonemura Y., Miwa K., Miyazaki I. (1992). Expression of p53 protein in colorectal cancer and its relationship to short-term prognosis.. Cancer.

